# Taking stress with much more than a pinch of salt: *osGRF7* regulates salinity resistance in rice through arbutin biosynthesis

**DOI:** 10.1093/plcell/koae139

**Published:** 2024-05-03

**Authors:** Christian Damian Lorenzo

**Affiliations:** Assistant Features Editor, The Plant Cell, American Society of Plant Biologists; Center for Plant Systems Biology, VIB, B-9052 Gent, Belgium; Department of Plant Biotechnology and Bioinformatics, Ghent University, B-9052 Gent, Belgium

Soil salinity is a major challenge for agricultural crops as it causes a severe reduction in growth, compromising biomass, seed size, and grain yield (van Zelm et al. 2020). To adapt to this salt stress, plants enhance the synthesis of metabolites that act as osmoprotectants (Fatima et al. 2021). For example, arbutin (also known as beta-arbutin) is a metabolite synthesized by some plant species that has been proposed to contribute to abiotic stress tolerance due to its antioxidant properties and ability to stabilize membranes (Oliver et al. 2001). How certain metabolites such as arbutin are connected to growth regulation is still a matter of research, but some molecular players have already been proposed. The *GROWTH REGULATOR FACTOR* (*GRF*) clade is a transcription factor family present in several plant species. Together with its cofactors, the *GRF-INTERACTING FACTORS* (*GIFs*), they regulate several developmental processes, such as organ growth, plant architecture determination, and abiotic stress responses, including salt stress (Liebsch and Palatnik 2020). However, how this latter role is mediated is yet unknown.

In this issue of *The Plant Cell*, **Chen and colleagues** (Chen et al. 2024) discovered that high levels of *OsGRF7 *s are associated with increased arbutin content and higher salt tolerance. The authors observed that rice lines overexpressing *OsGRF7* (*GRF7-OE*) had higher survival rates under high-salt conditions and increased grain size than control plants. In contrast, RNAi (*GRF7*-Ri) or genetic knockout (*grf7*) lines displayed an increased salt sensitivity. Untargeted and targeted metabolic profiling assays revealed that *GRF7*-OE had increased arbutin content. Since arbutin has not been directly associated to salt stress before, the group wanted to discover if the higher survival rates were related to arbutin increase. External application of arbutin to plants under high-salt concentrations restored the salt sensitivity of the *GRF7*-Ri and KO lines to wild-type levels, linking arbutin to salt stress tolerance. Arbutin action is speculated to be related to its antioxidative properties. As such, the research team observed that reactive oxygen species (ROS) levels were reduced in roots of *GRF7*-OE lines but increased in *GRF7*-Ri and *grf7*.

Going further into the details of how *OsGRF7* increases arbutin levels, Chen at al. found that 2 *URIDINE DIPHOSPHATE GLYCOSYLTRANSFERASES* (*UGT1* and *UGT5*) were upregulated in wild-type lines subjected to salt stress, as well as in *GRF7*-OE with or without salt stress. Considering UGTs are enzymes that catalyze the type of reactions necessary for arbutin biosynthesis, it was hypothesized that *OsGRF7* may induce their expression to promote arbutin biosynthesis. By means of electrophoretic mobility shift and transactivation assays, the group showed that *OsGRF7* indeed binds to and upregulates *UGT1* and *UGT5* expression. Moreso, heterologous assays performed in *E. coli* corroborated that these 2 enzymes catalyze arbutin biosynthesis. Consequently, KO lines of *UGT1* and *UGT5* in the *GRF7*-OE background showed reduced arbutin content, salt tolerance, and grain size, confirming that arbutin biosynthesis positively regulates these traits.

Finally, looking to expand the gene network around *OsGRF7* action, authors screened for OsGRF7 interactors through yeast 2-hybrid screenings and found an F-box-containing protein, *F-BOX CONTAINING PROTEIN 13* (*OsFBO13*). As F-Box proteins are important for protein degradation, they performed degradation assay and confirmed that OsGRF7 protein is indeed a substrate for *OsFBO13* and that their interaction leads to the degradation of OsGRF7. They also showed that OsFBO13 competes with OsGIFs (*GRFs* coactivator) to interact with OsGRF7; by use of overexpression (*FBO13*-OE) and KO (*fbo13*) lines, they demonstrated that this gene is a negative regulator of rice salt stress responses. The group forwarded a possible model of action for the *GRF7-FBO13-*arbutin module in the *FBO13-OE* and *fbo13* backgrounds (Fig.).

**Figure.**
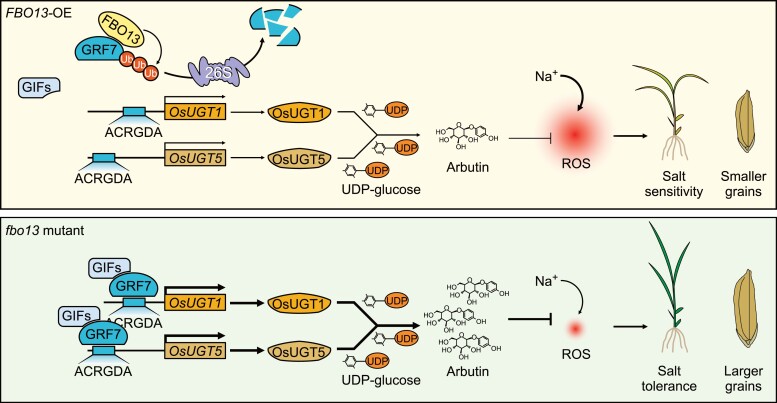
Model of action of *OsGRF7* regulating salt stress through arbutin biosynthesis in FBO13-OE and the *fbo13* mutant. *OsFBO13ox* (*left*) results in decreased arbutin content and salt sensibility while KO of it (right) leads to high arbutin content, low ROS, and increased salt tolerance and grain size. Reprinted from Chen et al. (2024), Figure 8.

In conclusion, these findings highlight a novel role for arbutin in salt tolerance coordinated by *OsGRF7* and *OsFBO13* with potential application in rice breeding.

## Dive Curated Terms

The following phenotypic, genotypic, and functional terms are of significance to the work described in this paper:

DIPHOSPHATE CHEBI: CHEBI:18361

uridine CHEBI: CHEBI:16704

GRF7 Gramene: Os12g0484900
GRF7 Araport: Os12g0484900

## References

[koae139-B1] Chen Y , Dan Z, Li S. GROWTH REGULATING FACTOR 7-mediated arbutin metabolism enhances rice salt tolerance. Plant Cell. 2024:36 (8):2834–2850. 10.1093/plcell/koae140 PMC11289636 38701348

[koae139-B2] Fatima A , Singh G, Patel A, Tiwari S, Gupta D, Prajapati DK, Dubey A, et al Effects of salt stress on osmolyte metabolism of crop plants and mitigating strategy by osmolyte. In: Singh P, Singh M, Singh RK, Prasad SM, editors. Physiology of salt stress in plants. John Wiley & Sons, Ltd; 2021. p. 177–197. 10.1002/9781119700517.ch10.

[koae139-B3] Liebsch D , Palatnik JF. MicroRNA miR396, GRF transcription factors and GIF co-regulators: a conserved plant growth regulatory module with potential for breeding and biotechnology. Curr Opin Plant Biol. 2020:53:31–42. 10.1016/j.pbi.2019.09.008 31726426

[koae139-B4] Oliver AE , Hincha DK, Tsvetkova NM, Vigh L, Crowe JH. The effect of arbutin on membrane integrity during drying is mediated by stabilization of the lamellar phase in the presence of nonbilayer-forming lipids. Chem Phys Lipids. 2001:111(1):37–57. 10.1016/S0009-3084(01)00141-4 11438283

[koae139-B5] van Zelm E , Zhang Y, Testerink C. Salt tolerance mechanisms of plants. Ann. Rev. Plant Biol. 2020:71(1):403–433. 10.1146/annurev-arplant-050718-100005 32167791

